# Entomopathogenic Nematodes and Bioactive Compounds of Their Bacterial Endosymbionts Act Synergistically in Combination with Spinosad to Kill *Phthorimaea operculella* (Zeller, 1873) (Lepidoptera: Gelechiidae), a Serious Threat to Food Security

**DOI:** 10.3390/microorganisms13102368

**Published:** 2025-10-15

**Authors:** Ebubekir Yüksel, Rachid Lahlali, Aydemir Barış, Muhammad Sameeullah, Furkan Ulaş, Abdurrahman Sami Koca, Essaid Ait Barka, Mustafa İmren, Abdelfattah Dababat

**Affiliations:** 1 Department of Plant Protection, Faculty of Agriculture, Erciyes University, 38030 Kayseri, Türkiye; ebubekiryuksel@erciyes.edu.tr; 2 Phytopathology Unit, Department of Plant Protection, Ecole National of Agriculture Meknes, Km 10, Rte Haj Kaddour, BP S/40, Meknes 50001, Morocco; 3 Plant Protection Central Research Institute, Gayret Neighborhood, Fatih Sultan Mehmet Boulevard, 06172 Ankara, Türkiye; aydemirbaris02@gmail.com; 4 Department of Field Crops, Faculty of Agriculture, Bolu Abant Izzet Baysal University, 14030 Bolu, Türkiye; muhammad.sameeullah@ibu.edu.tr; 5 Department of Plant Protection, Faculty of Agricultural Sciences and Technologies, Sivas University of Science and Technology, 58010 Sivas, Türkiye; furkanulas@sivas.edu.tr; 6 Department of Plant Protection, Faculty of Agriculture, Bolu Abant Izzet Baysal University, 14030 Bolu, Türkiye; a.samikoca@yahoo.com.tr (A.S.K.); mustafaimren@ibu.edu.tr (M.İ.); 7 Induced Resistance and Plant Biosection Research Unit, EA 4707-USC INRAE1488, Reims Champagne-Ardenne University, 51687 Reims, France; 8 International Maize and Wheat Improvement Centre (CIMMYT), 06170 Ankara, Türkiye; a.dababat@cgiar.org; 9 Faculty of Agriculture, The University of Jordan, Amman 11942, Jordan

**Keywords:** staple food, sustainable control, synergistic interaction, symbiotic bacteria

## Abstract

As a staple food, potato (*Solanum tuberosum* L.) (Solanaceae) is one of the most produced food crops to ensure food security. The potato tuber moth (PTM), *Phthorimaea operculella* (Zeller, 1873) (Lepidoptera: Gelechiidae), is a major pest of potato, damaging both the growing and storage processes. In recent years, green pest control strategies have been gaining importance to reduce the adverse effects of chemicals and protect the environment. Entomopathogenic nematodes (EPNs) and their bacterial endosymbionts (*Xenorhabdus* and *Photorhabdus* spp.) have been one of the top topics studied in sustainable pest control approaches. In the present study, the two most common EPN species, *Steinernema feltiae* and *Heterorhabditis bacteriophora*, and their bacterial associates, *Xenorhabdus bovienii* and *Photorhabdus luminescens* subsp. *kayaii* were evaluated against PTM larvae separately and in combination with spinosad. The survival rates of infective juveniles (IJs) of EPNs were over 92% after 72 h of direct exposure to spinosad. Co-application of EPNs and bioactive compounds (BACs) of endosymbiotic bacteria with spinosad induced synergistic interactions and achieved the maximum mortality (100%) in PTM larvae 48 h post-treatment. Spinosad and BAC combinations were highly efficient in controlling the PTM larvae and provided LT_50_ values below 23.0 h. Gas chromatography mass spectrometry (GC-MS) analysis identified 29 compounds in total, 20 of which belonged to *P. luminescens* subsp. *kayaii*. The results indicate that the integration of EPNs and BACs of endosymbiotic bacteria with spinosad presents a synergistic interaction and enhances pest control efficacy.

## 1. Introduction

Potato (*Solanum tuberosum* L.) (Solanaceae) is the most produced non-cereal staple crop across the globe, which plays a major role in reducing poverty as a crucial food supply for many people [1]. However, there has been a 5% decrease in the world potato planting area in the past 10 years [1]. In addition, potato productivity is projected to decline in the coming decades in many parts of the world due to the impact of climate change on agricultural pests and diseases, as well as extreme weather events [2,3,4]. Therefore, crop losses associated with pests and diseases should be minimized to ensure food supply and food security. The potato tuber moth (PTM), *Phthorimaea operculella* (Zeller, 1873) (Lepidoptera: Gelechiidae), is a global pest of potato plants that has been reported in temperate, sub-tropical, and tropical zones [5,6,7,8]. The larvae of PTM inflict serious damage to potato plants and tubers both in the field and storage conditions, and, therefore, are a major concern for growers [8]. Although larvae of PTM can feed on different parts of potato plants, including stems and leaves, tuber damage caused by larvae in storage is of major economic importance due to the quality deterioration [9]. In poor storage conditions, PTM larvae can cause serious post-harvest losses varying between 50% and 100% in stored potatoes [5,10].

Today, the control of PTM relies mainly on synthetic insecticide treatments [11]. However, due to the cryptic feeding habits of larvae and the decreased susceptibility of PTM populations to chemicals [12], synthetic insecticides generally fail to provide adequate protection against PTM [13]. Additionally, excessive use of chemicals seriously harms the environment and human health [14]. Therefore, over the last decade, green alternatives to chemicals such as entomopathogenic nematodes (EPNs) (Rhabditida: Steinernematidae and Heterorhabditidae) have gained great importance in PTM control to reduce reliance on synthetic insecticides [15,16,17].

EPNs are obligate soil-borne parasites of insects that are able to suppress pest populations in a short period of time. The infective juveniles (IJs) of EPNs are non-feeding and only stage with active host-seeking ability in soil [18]. They act as specific vectors of their endosymbiotic bacteria (*Xenorhabdus* and *Photorhabdus* spp.), which are mainly responsible for the production of toxins that kill insect hosts [19]. Once penetrating an insect host, the IJs transmit their bacterial symbionts into the host hemolymph, and the bacteria induce the pathogenicity process by releasing a range of bioactive compounds (BACs) which exhibit insecticidal and immunosuppressive activities [20,21]. This process eventually results in the death of many host insects in 24–48 h [22,23,24].

Recent studies have highlighted that the in vitro produced BACs of endosymbiotic bacteria are also lethal to some insects upon contact or oral treatments [25,26,27,28]. However, to the author’s knowledge, no studies have investigated the toxicity of BACs produced by endosymbiotic bacteria of EPNs, and only several studies evaluated the biocontrol potential of EPNs against PTM [16,17,29,30]. In addition, the compatibility and synergistic relationship of biocontrol agents with biopesticides such as spinosad have been the topic of recent studies and have provided a new perspective on the integrated biocontrol of agricultural pests [3,9,13]. Spinosad is one of the most used microbial insecticides produced from *Saccharopolyspora spinosa*, a soil-borne bacterium [17,31]. Spinosad contains toxins called spinosyns that are toxic to the nervous system of insects upon oral or contact applications, and combined applications of spinosad with other bioproducts may contribute to pest control strategies [17,31]. Therefore, the present study was conducted to offer a holistic approach to the biocontrol of PTM, evaluating the two most common EPNs and their BACs of endosymbiotic bacteria alone and in combination with spinosad.

## 2. Materials and Methods

### 2.1. Entomopathogenic Nematodes (EPNs)

The bioassays were performed using two most common EPN species, *Steinernema feltiae* (MCB-8) and *Heterorhabditis bacteriophora* (AVB-15), isolated from the Cappadocia region of Türkiye (Table 1). The IJs of these EPNs were routinely cultivated in vivo using the larvae of *Galleria mellonella* (L.) (Lepidoptera: Pyralidae) under laboratory conditions (25 ± 1 °C, relative humidity of 60 ± 5%). Newly emerged IJs were stored in distilled water for two weeks at 9 ± 1 °C until bioassays. *G. mellonella* larvae were obtained from the stock culture of the Entomology Laboratory of Erciyes University and were reared at a temperature of 30 ± 1 °C and relative humidity of 65 ± 5% under 12 h light and dark conditions with an artificial diet [31].

### 2.2. Potato Tuber Moth (PTM)

The larvae of PTM were obtained from the stock culture of Directorate of Plant Protection, Central Research Institute, and reared on potato tubers in cages (60 × 60 × 60 cm) under controlled conditions (25 ± 1 °C, relative humidity of 65 ± 5%, and photoperiod of 16L: 8D). A 10% honey-water (*v*/*v*) solution were provided for the adults as food supplement. After two generations, the last instar larvae of PTM were collected using a fine paintbrush.

### 2.3. Survival of EPNs in Spinosad

The lethal effect of Spinosad (LASER™, 480 g/L) (Dow Agrosciences Ltd., Indianapolis, IN, USA) on the EPNs was determined by exposing 100 IJs of each EPN species to 1 mL of spinosad solution prepared at the highest recommended field concentration (30 mL/100 L water). The experiment was conducted in 24-well plates containing 100 IJs/10 μL and 1 mL of spinosad solution. As control treatment, double-distilled water was used. The well-plates were maintained in an orbital shaker at 100 rpm shaking frequency at 25 ± 1 °C in darkness. A 100 μL sample was retrieved daily from each well to check the survival of IJs under a stereomicroscope (Leica M125, Leica Microsystems, Deerfield, IL, USA). The experiments consisted of four replications (10 wells) and were repeated twice.

### 2.4. Pathogenicity Bioassay with EPNs and Spinosad

The pathogenicity bioassays of EPNs alone and in combination with spinosad solution were carried out in Petri plates (90 mm Ø) with two filter papers at the bottom. Ten healthy 4th instar larvae of PTM were transferred into plates, and 1 mL of 200 IJs/mL tap water, spinosad (30 mL/100 L water), and IJs-spinosad solution containing 200 IJs of each EPN species (200 IJs/10 μL + 990 μL spinosad) was inoculated via micropipetting. Tiny potato pieces were provided for the larvae as food and were replaced daily with a new batch. The plates were covered with parafilm and maintained at 25 ± 1 °C and a relative humidity of 65 ± 5%. The PTM larvae were checked daily, and dead larvae were recorded during the bioassay. In the control group, double-distilled water was used. Each treatment consisted of 4 replicates with 10 larvae per replicate, and the whole bioassay was conducted twice.

### 2.5. Isolation of Endosymbiotic Bacteria

The bacterial associates of EPNs were isolated from newly harvested 1000 IJs of each EPN species [24]. These IJs were surface-sterilized using 10% sodium hypochlorite (NaOCl) (10% *w*/*vol*) solution, and were rinsed three times in sterile phosphate-buffered saline (PBS). The endosymbiotic bacteria were extracted by crushing the IJs in 1 mL of PBS using a sterile pestle. Then, 50 μL of crushed IJs-PBS solution was spread onto Petri plates (90 mm Ø) containing nutrient agar supplemented with 0.025 g bromothymol blue (Labchem, Greenford, UK) and 0.004 g triphenyl tetrazolium chloride (NBTA medium) (Sigma Chemical, St. Louis, MO, USA) [33]. The Petri plates were incubated for bacterial colony formation at 25 ± 1 °C, relative humidity of 40% in the dark for 48 h. Bacterial colonies that appeared circular and blue-greenish were subcultured on NBTA medium. This step was repeated several times to ensure the purity of bacterial colonies [33].

### 2.6. Preparation of Bioactive Compounds (BACs)

One isolated bacterial colony was picked from each strain with a sterile loop and inoculated into 100 mL of Luria–Bertani (LB) broth in a 250 mL Erlenmeyer flask. Then, the flasks were further cultivated in a shaker (180 rpm) for 6 days in a growth chamber at 28 ± 1 °C, relative humidity of 65 ± 5%, and in full darkness [19,33]. Following the incubation period, the supernatants containing the BACs were separated by filtering through a 0.22 μm millipore filter (Thermo Scientific, New York, NY, USA) after centrifuging the bacterial suspensions in a refrigerated centrifuge in 50 mL conical centrifuge tubes at 20,000 rpm for 15 min at 4 °C. To ensure the purity of the supernatant solutions, the filtered suspension was re-inoculated into NBTA plates and incubated under the aforementioned conditions.

### 2.7. Bioassay with BACs and Spinosad

Insecticidal activity of BACs in the cell-free supernatant solutions (CFSs) was determined by direct contact application on 4th instar larvae of PTM under controlled conditions. Ten larvae of PTM were transferred into Petri plates (90 mm Ø) lined with two filter papers and exposed to 1 mL of CFS, spinosad (30 mL/100 L water), and a mixture of 50% spinosad and 50% (*v*/*v*) CFS. To avoid larval starvation, tiny potato pieces were given to larvae as a food source and replaced daily with a new batch. The plates were incubated at 25 ± 1 °C with a relative humidity of 65 ± 5% after being sealed with parafilm. The larvae were checked daily, and mortality rates were noted. Control groups consisted of spray application of pure water and nutrient broth (NB) following the same procedure. Each treatment consisted of 4 replicates with 10 larvae per replicate, and the whole bioassay was conducted twice.

### 2.8. Gas Chromatography Mass Spectrometry (GC-MS) Analysis

To determine the BACs of endosymbiotic bacteria, the particle-free supernatant solutions (1 μL) were subjected to GC-MS analysis using Shimadzu GC-MS/QP2010 ULTRA fitted with Rtx-5MS non-polar capillary column (Benner Circle, Bellefonte, PA, USA), which had a length of 30 m × 0.25 mm inner diameter × 0.25 µm film thickness. The injection was performed in splitless mode for 1 min using helium (99.99%) as the carrier gas. The solutions were injected at a split ratio of 1/50. The oven temperature was programmed from 40 °C (held for 1 min) to 280 °C (held for 5 min), and the interface temperature was 100 °C (held for 1 min). The column flow was 2 mL/min with a linear velocity. Scan ranges were set at 45–650 *m*/*z* with a rate of 0.3 scans. Ion source and interface temperatures were 300 and 280 °C, respectively. The electron ionization (EI) was at 70 eV. The BACs were identified by comparing the mass spectra of the BACs (≥95%) with those in the database of NIST Mass Spectral Library (http://nistmassspeclibrary.com, accessed on 23 May 2025).

### 2.9. Statistical Analysis

The data were analyzed using IBM^®^ SPSS Statistics 22.0 software (SPSS Inc., Chicago, IL, USA) after arcsine transformations (square root) were performed on the percentage of mortality of PTM larvae and IJs of EPNs. The survival data of EPNs in spinosad solutions were analyzed by one-way ANOVA. The mortality data of PTM were subjected to two-way ANOVA. Means were compared using the Tukey test at a *p* = 0.05 level (*p* ≤ 0.05). To estimate the 50% lethal time (LT_50_) values for each application, the mortality data obtained at different times were subjected to probit analysis [34].

## 3. Results

### 3.1. Survival of EPNs in Spinosad

Spinosad application had a significant effect on the survival of IJs compared to control treatments. The IJs of *H. bacteriophora* AVB-15 generally exhibited higher survival than *S. feltiae* MCB-8. However, the decrease in the survival of IJs of both EPN species was negligible and did not exceed 8% after 72 h of direct exposure (Table 2 and Table 3).

### 3.2. Pathogenicity Bioassay with EPNs and Spinosad

The analysis indicated that the treatments and exposure time had a significant effect on the mortality of PTM larvae (Table 4). The spinosad combinations of EPNs remarkably increased the larval mortality compared to the control group, spinosad, and EPN applications alone. However, *H. bacteriophora* AVB-15 and spinosad combination yielded the highest mortality result after 24 h of exposure. Remarkably, only spinosad and its combination with EPNs achieved the maximum mortality (100%) on PTM larvae 48 h after treatment. After 72 h of application, all treatments caused the maximum mortality (100%) except for *S. feltiae* MCB-8 (Table 5). The estimated LT_50_ values for PTM larvae ranged from 23.52 to 57.84 hrs, with spinosad yielding the lowest estimated LT_50_ value (Table 6).

### 3.3. Bioassay with BACs and Spinosad

The mortality of PTM larvae differed significantly among CFSs containing BACs and spinosad treatments, along with their combinations (Table 7). Similarly to EPN treatments, spinosad combinations of CFSs led to higher mortalities on PTM larvae compared to CFS and spinosad treatments alone, and the maximum mortality (100%) was achieved after 48 h of exposure. Although the application of CFSs of *X. bovienii* MCB-8 alone did not exceed 50% mortality on PTM larvae, the larval mortality reached 80% after 72 h of exposure (Table 8).

Unlike the EPN-spinosad bioassay, the lowest LT_50_ values were obtained from spinosad and CFS combinations. The LT_50_ values were less than 24 h for spinosad treatment alone and its combinations with CFSs. The LT_50_ value of CFSs of *P. luminescens* subsp. *kayaii* AVB-15 was notably lower than the CFSs of *X. bovienii* MCB-8 (Table 9).

### 3.4. GC-MS Analysis

The GC-MS chromatogram of the CFSs of endosymbiotic bacteria revealed the presence of 9 and 20 BACs for *X. bovienii* MCB-8 and *P. luminescens* subsp. *kayaii* AVB-15, resptectively. The compounds are presented in Table 10 and Table 11. Two major compounds, namely Pyrrolo[1,2-a]pyrazine-1,4-dione, hexahydro-3-(phenylmethyl)- and oxime- methoxy-phenyl-_, were common in the CFSs of both endosymbiotic bacteria.

## 4. Discussion

The integration and co-application of biocontrol agents with biopesticides is a practical and sustainable approach and enables a cost-effective and time-saving pest control. However, the compatibility of EPNs with chemicals varies depending on species/isolates [35,36]. Therefore, chemicals need to be assessed for the potential toxicity on the survival and infectivity of EPNs before tank-mixing. Spinosad is a broad-spectrum bio-insecticide derived from the fermentation of the soil bacterium *Saccharopolyspora spinosa* and has been successful in the control of a wide range of insect pests [37]. The compatibility of some EPN species with agrochemicals was studied in earlier studies, and EPNs exhibited a high tolerance to spinosad in short-term exposures, while being adversely affected by synthetic chemicals [38,39]. In parallel with our results, Rashad et al. [40] highlighted that *Steinernema carpocapse* and *Heterorhabditis indica* were highly tolerant to spinosad (Tracer^®^ 240SC) applications, and the survival of EPNs was over 85% after 48 h of exposure. In the present study, although both EPN species used were found to be highly tolerant, with a survival rate of over 93%, *H. bacteriophora* AVB-15 exhibited a higher tolerance to spinosad treatment than *S. feltiae* MCB-8 (Table 3). These results are in agreement with the findings of Kasi et al. [41] and Yüksel and Canhilal [39]. The higher tolerance of *H. bacteriophora* to spinosad treatments can be associated with the longer retention of the second-stage cuticle by the IJs of *H. bacteriophora* compared to *S. feltiae* [42]. This may have provided an extra protection for *H. bacteriophora* against the spinosad. However, in some cases, the interaction of EPNs with chemicals may be antagonistic or synergistic depending on EPN species/isolates and chemicals used [36,43,44]. Our results also revealed that a synergistic interaction occurred between spinosad combinations of both EPNs and bacterial metabolites of their endosymbiotic bacteria against the PTM larvae. Similarly, Abdel-Razek and Abd-Elgawad [45] reported that *Steinernema riobrave* combined with spinosad led to higher mortalities on the larvae of *Ceratitis capitata* (W.) (Diptera: Tephritidae) in laboratory and field experiments compared to EPNs and spinosad treatments alone. In another study, *Steinernema carpocapsae* and *Heterorhabditis indica* displayed a synergistic effect with spinosad against the larvae of *Spodoptera litura* (F.) (Lepidoptera: Noctuidae) [40]. The synergistic effect of spinosad with EPNs and their bacterial metabolites could be directly related to the mode of action mechanism. Spinosad disrupts the neural mechanism of insects, leading to involuntary muscle spasms and paralysis [46], which may have facilitated the penetration and invasion of IJs of EPNs into the host body through natural body openings without physical resistance [47]. Another possible explanation for the synergistic effect could be associated with the insect’s immune response. Earlier studies indicated that spinosad causes immune system abnormalities in target insects by altering specific immune functions, which render host insects more vulnerable to infection by EPNs [47,48,49]. Similarly, EPNs and their bacterial associates also suppress immunoresponses of host insects through inhibition of eicosanoid biosynthesis, which modulates melanotic encapsulation and phagocytosis [50,51,52]. Thus, PTM larvae may have failed to overcome the nematode infection under the pressure of two different immunosuppressive activities of EPNs and spinosad. However, Chandra et al. [52] suggested that the pathogenicity and immunosuppression ability of EPNs depend on their symbiotic bacteria, which could be a significant factor affecting the insect mortality by different EPN species and the metabolites of their symbiotic bacteria. After being vectored into host hemolymph by IJs, *Xenorhabdus* and *Photorhabdus* spp. bacteria produce a wide range of bioactive compounds, including toxin complexes, peptides, proteases, and lipases [22,53]. However, the composition of these metabolites and their immunosuppressive activities vary greatly among symbiotic bacteria species and strains, leading to various mortalities in host insects. For example, Hasan et al. [54] reported a significant variation in the chemical composition of organic extracts produced by different *Xenorhabdus nematophila* strains. In the present study, GC-MS analysis identified 29 compounds, 2 of which were found in the CFSs of both symbiotic bacteria. In earlier studies, among these compounds, 2-Pyrrolidone and 2-Piperidone in varying ratios were commonly reported for the *Xenorhabdus* and *Photorhabdus* spp. bacteria [21,27,55]. Although the exact role of most of these compounds on insects remains unknown, the bioactivity of some of these compounds, such as Pyrrolo [1, 2-a] pyrazine-1, 4-dione, hexahydro-3-(phenylmethyl)- and Pyrrolo(1,2-a)pyrazine-1,4-dione, hexahydro-3-(methylethyl)- has been elucidated as antibacterial and antifungal, respectively [56,57]. It is reasonable to speculate that most of these compounds play an important role in the pathogenicity and immunosuppression of host insects and perform insecticidal and antimicrobial activity, as suggested in earlier studies. For example, 1,2-benzenedicarboxylic and n-Decanoic acids, and 3-Benzylidene-hexahydro-pyrrolo derivatives have been reported to have an insecticidal and antimicrobial activity against *G. mellonella* larvae [26,27,54,55,58]. In addition, these results, along with earlier studies, also highlighted that the BACs of *Xenorhabdus* and *Photorhabdus* spp. bacteria are toxic against insects in contact applications, which clearly shows that these compounds can be absorbed by the insect cuticle and natural openings (Stigma, anus, etc.) through absorption, reaching target sites inside the insect [15,32,59,60]. Some of these BACs may have different roles and activities in insect mortalities. However, further studies with these compounds will provide valuable insights into their bioactivity.

## 5. Conclusions

This study evaluated the control potential of EPNs and the BACs of their endosymbiotic bacteria on PTM larvae separately and in combination with spinosad. Our findings revealed that the EPNs and the BACs of their endosymbionts had great potential in the control of PTM. However, the combined application of EPNs and the BACs of their endosymbiotic bacteria with spinosad achieved more efficient pest control than separate applications. Moreover, in this study, the chemical composition of the BACs of their endosymbiotic bacteria was also explored, some of which are responsible for the insecticidal and immunosuppressive activity of *Xenorhabdus bovienii* and *Photorhabdus luminescens* subsp. *kayaii* bacteria. However, further studies are needed to better understand the bioinvolvement of these compounds in the pathogenicity and immunosuppressive activity process.

## Figures and Tables

**Table 1:** Entomopathogenic nematodes (EPNs) and their endosymbiotic bacteria (ESB) used in the bioassays.

EPNs	GenBank Accession Number	Reference	ESB	GenBank Accession Number	Reference
*Steinernema feltiae* MCB-8	MG602334	[32]	*Xenorhabdus bovienii* MCB-8	MW403818	[24]
*Heterorhabditis bacteriophora* ABV-15	MG602333	[32]	*Photorhabdus luminescens* subsp. *kayaii* ABV-15	MG602333	[24]

**Table 2:** One-way ANOVA analysis of the survival of entomopathogenic nematodes in spinosad.

	Degree of Freedom	F Value	*p* Value
**24 h**	1	13,000	0.002
**48 h**	1	45,500	<0.001
**72 h**	1	108,273	<0.001

**Table 3:** The survival rates of infective juveniles (IJs) of entomopathogenic nematodes (EPNs) after exposure to spinosad (LASER™ 480 SC).

Exposure Time	Treatments *	Survival Rates (Mean % ± Std. Error) *
24 h	CONTROL_PW	100.0 ± 0.0 A
	*Steinernema feltiae* MCB-8	98.0 ± 0.4 B
	*Heterorhabditis bacteriophora* AVB-15	99.5 ± 0.2 A
48 h	CONTROL_PW	99.2 ± 0.4 A
	*Steinernema feltiae* MCB-8	94.5 ± 0.2 C
	*Heterorhabditis bacteriophora* AVB-15	97.5 ± 0.2 B
72 h	CONTROL_PW	98.5 ± 0.2 A
	*Steinernema feltiae* MCB-8	92.7 ± 0.2 C
	*Heterorhabditis bacteriophora* AVB-15	95.5 ± 0.2 B

* PW: Pure water. Capital letters indicate that statistically significant differences were observed among different exposure times (Tukey; *p* ≤ 0.05).

**Table 4:** Two-way ANOVA analysis of entomopathogenic nematodes, spinosad, and their combinations, along with control treatments (Nutrient Broth and Pure water).

	Degree of Freedom	F Value	*p* Value
**Treatments (T)**	6	576.331	<0.001
**Exposure Time (t)**	2	229.145	<0.001
**T × t**	12	17.596	<0.001
**Error-1**	18		
**Error-2**	36		

**Table 5:** The insecticidal effects of entomopathogenic nematodes (200 IJs/mL water), spinosad, and their combinations on *Phthorimaea operculella* (Zeller, 1873) (Lepidoptera: Gelechiidae) larvae after different exposure times (24, 48, and 72 h).

Exposure Time	Treatments *	Mortality Rates (Mean % ± Std. Error) *
24 h	CONTROL_PW	0.00 ± 0.0 Aa
	*Steinernema feltiae* MCB-8	32.50 ± 4.7 Ba
	*Heterorhabditis bacteriophora* AVB-15	67.50 ± 4.7 Ca
	Spinosad	55.00 ± 2.8 Ca
	Spinosad + *Steinernema feltiae* MCB-8	65.00 ± 2.8 Ca
	Spinosad + *Heterorhabditis bacteriophora* AVB-15	82.50 ± 2.5 Da
48 h	CONTROL_PW	0.00 ± 0.0 Aa
	*Steinernema feltiae* MCB-8	55.00 ± 2.8 Bb
	*Heterorhabditis bacteriophora* AVB-15	87.50 ± 4.7 Cb
	Spinosad	100.00 ± 0.0 Db
	Spinosad + *Steinernema feltiae* MCB-8	100.00 ± 0.0 Db
	Spinosad + *Heterorhabditis bacteriophora* AVB-15	100.00 ± 0.0 Db
72 h	CONTROL_PW	0.00 ± 0.0 Aa
	*Steinernema feltiae* MCB-8	80.00 ± 4.0 Bc
	*Heterorhabditis bacteriophora* AVB-15	100.00 ± 0.0 Cc
	Spinosad	100.00 ± 0.0 Cb
	Spinosad + *Steinernema feltiae* MCB-8	100.00 ± 0.0 Cb
	Spinosad + *Heterorhabditis bacteriophora* AVB-15	100.00 ± 0.0 Cb

* PW: Pure water. Capital letters indicate significant differences among different treatments for the same exposure time. Small letters indicate significant differences for the same treatment at different exposure times (Tukey; *p* ≤ 0.05).

**Table 6:** Comparison of lethal times (LT_50_) of entomopathogenic nematodes, spinosad, and their combinations on *Phthorimaea operculella* (Zeller, 1873) (Lepidoptera: Gelechiidae) larvae.

Probit Analysis	*Steinernema feltiae* MCB-8	*Heterorhabditis bacteriophora* AVB-15	Spinosad	Spinosad + *Steinernema feltiae* MCB-8	Spinosad + *Heterorhabditis bacteriophora* AVB-15
n	40	40	40	40	40
X^2^	5.865	0.105	0.010	0.196	0.586
df	1	1	1	1	1
Slope ± SE	8.25 ± 1.5	15.19 ± 9.1	12.01 ± 7.1	11.50 ± 5.1	11.45 ± 5.1
LT_50_ (h)	57.8	45.3	23.5	42.7	44.6

**Table 7:** Two-way ANOVA analysis of cell-free supernatants of endosymbiotic bacteria, spinosad, and their combinations, along with control treatments (Nutrient Broth and Pure water).

	Degree of Freedom	F Value	*p* Value
Treatments (T)	6	252.130	<0.001
Exposure Time (t)	2	178.913	<0.001
T × t	12	30.381	<0.001
Error-1	21		
Error-2	42		

**Table 8:** The insecticidal effects of cell-free supernatants of endosymbiotic bacteria, spinosad, and their combinations on *Phthorimaea operculella* (Zeller, 1873) (Lepidoptera: Gelechiidae) larvae after different exposure times (24, 48, and 72 h).

Exposure Time	Treatments *	Mortality Rates (Mean% ± Std. Error) *
24 h	CONTROL_NTBT	2.50 ± 2.5 Aa
	CONTROL_PW	0.00 ± 0.0 Aa
	CFSs of *Xenorhabdus bovienii* MCB-8	35.00 ± 2.8 Ba
	CFSs of *Photorhabdus luminescens* subsp. *kayaii* AVB-15	22.50 ± 2.5 Ba
	Spinosad	55.00 ± 2.8 Ca
	Spinosad + CFSs of *Xenorhabdus bovienii* MCB-8	92.50 ± 2.5 Da
	Spinosad + CFSs of *Photorhabdus luminescens* subsp. *kayaii* AVB-15	77.50 ± 2.5 CDa
48 h	CONTROL_NTBT	2.50 ± 2.5 Aa
	CONTROL_PW	0.00 ± 0.0 Aa
	CFSs of *Xenorhabdus bovienii* MCB-8	47.50 ± 4.7 Ba
	CFSs of *Photorhabdus luminescens* subsp. *kayaii* AVB-15	87.50 ± 6.2 Cb
	Spinosad	100.00 ± 0.0 Db
	Spinosad + CFSs of *Xenorhabdus bovienii* MCB-8	100.00 ± 0.0 Da
	Spinosad + CFSs of *Photorhabdus luminescens* subsp. *kayaii* AVB-15	100.00 ± 0.0 Db
72 h	CONTROL_NTBT	2.50 ± 2.5 Aa
	CONTROL_PW	0.00 ± 0.0 Aa
	CFSs of *Xenorhabdus bovienii* MCB-8	80.00 ± 9.1 Bb
	CFSs of *Photorhabdus luminescens* subsp. *kayaii* AVB-15	90.00 ± 7.0 BCb
	Spinosad	100.00 ± 0.0 Cb
	Spinosad + CFSs of *Xenorhabdus bovienii* MCB-8	100.00 ± 0.0 Ca
	Spinosad + CFSs of *Photorhabdus luminescens* subsp. *kayaii* AVB-15	100.00 ± 0.0 Ca

* PW: Pure water; NTBT: Nutrient Broth. CFSs: cell-free supernatants of endosymbiotic bacteria. Capital letters indicate significant differences among different treatments for the same exposure time. Small letters indicate significant differences for the same treatment at different exposure times (Tukey; *p* ≤ 0.05).

**Table 9:** Comparison of lethal times (LT_50_) of cell-free supernatants of endosymbiotic bacteria, spinosad, and their combinations on *Phthorimaea operculella* (Zeller, 1873) (Lepidoptera: Gelechiidae) larvae.

Probit Analysis	CFSs of *Xenorhabdus bovienii* MCB-8	CFSs of *Photorhabdus luminescens* subsp. *kayaii* AVB-15	Spinosad	Spinosad + CFSs of *Xenorhabdus bovienii* MCB-8	Spinosad + CFSs of *Photorhabdus luminescens* subsp. *kayaii* AVB-15
n	40	40	40	40	40
X^2^	3.471	12.494	0.010	0.007	0.001
df	1	1	1	1	1
Slope ± SE	10.82 ± 2.5	4.96 ± 0.7	12.01 ± 7.1	10.46 ± 16.3	12.30 ± 31.5
LT_50_ (h)	62.3	32.7	23.5	22.7	21.2

**Table 10:** Identified bioactive compounds (BACs) from cell-free supernatants (CFSs) of *Xenorhabdus bovienii* MCB-8 endosymbiotic bacteria in GC-MS analysis.

Peak No.	Retention Time	Area	Area %	Compound Name	Formula
1	6.600	23,272	0.36	1,2-Propanediol, 3-methoxy-	C_4_H_10_O_3_
2	6.749	710,132	10.86	oxime- methoxy-phenyl-_	C_8_H_9_NO_2_
3	10.198	87,966	1.35	1-propanol 2-(2-hydroxypropoxy)-	C_6_H_14_O_3_
4	15.386	56,266	0.86	Isosorbide	C_6_H_10_O_4_
5	18.519	44,538	0.68	2,6-Diaminopyridine	C_5_H_7_N_3_
6	40.794	381,292	5.83	3-Methyl-1,4-diazabicyclo[4.3.0]nonan-2,5-dione, N-acetyl-	C_10_H_14_N_2_O_3_
7	46.364	2,364,240	36.15	Pyrrolo(1,2-a)pyrazine-1,4-dione, hexahydro-3-(methylethyl)-	C_10_H_16_N_2_O_2_
8	52.187	536,998	8.21	5,10-Diethoxy-2,3,7,8-tetrahydro-1H,6H-dipyrrolo[1,2-a:1′,2′-d]pyrazine	C_14_H_22_N_2_O_2_
9	71.998	2,334,572	35.70	Pyrrolo[1,2-a]pyrazine-1,4-dione, hexahydro-3-(phenylmethyl)-	C_14_H_16_N_2_O_2_

**Table 11:** Identified bioactive compounds (BACs) from cell-free supernatants (CFSs) of *Photorhabdus luminescens* subsp. *kayaii* AVB-15 endosymbiotic bacteria in GC-MS analysis.

Peak No.	Retention Time	Area	Area %	Compound Name	Formula
1	5.528	202,857	1.96	Butanoic acid 2-methyl-	C_5_H_10_O_2_
2	5.748	52,368	0.51	Pyridine, 2,3,4,5-tetrahydro-	C_5_H_9_N
3	6.067	83,622	0.81	Butanoic acid, 3-methyl-	C_5_H_10_O_2_
4	6.977	187,044	1.81	Oxime- methoxy-phenyl-_	C_8_H_9_NO_2_
5	8.587	38,398	0.37	1-Propanol, 3-(methylthio)-	C_4_H_10_OS
6	10.045	183,021	1.77	D-Pantolactone	C_6_H_10_O_3_
7	11.005	475,043	4.60	2-Pyrrolidone	C_4_H_7_NO
8	12.121	32,764	0.32	2-Phenylethanol	C_8_H_10_O
9	14.374	373,871	3.62	2-Piperidone	C_5_H_9_NO
10	14.711	102,016	0.99	Pyridine, 1-acetyl-1,2,3,4-tetrahydro-	C_7_H_11_NO
11	18.969	54,963	0.53	1H-Indole	C_8_H_7_N
12	19.685	882,574	8.54	3-aminopiperidine-2-one	C_5_H_10_N_2_O
13	23.101	66,976	0.65	Ethyl pipecolinate	C_8_H_15_NO_2_
14	33.446	91,362	0.88	2-Heptene, 5-ethyl-2,4-dimethyl-	C_11_H_22_
15	40.831	727,054	7.04	3-Methyl-1,4-diazabicyclo[4.3.0]nonan-2,5-dione, N-acetyl-	C_10_H_14_N_2_O_3_
16	43.377	2,815,688	27.26	Pyrrolo[1,2-a]pyrazine-1,4-dione, hexahydro-	C_7_H_10_N_2_O_2_
17	46.324	1,673,015	16.20	1,4-diaza-2, 5-dioxo-3-isobutyl bicyclo[4.3.0]nonane	C_11_H_18_N_2_O_2_
18	52.180	768,386	7.44	3,9-Dihydroxy-1,7-diazatricyclo[7.3.0.03,7]dodecane-2,8-dione	C_10_H_14_N_2_O_4_
19	66.868	129,920	1.26	Pyrrolo[1,2-a]pyrazine-3-propanamide, 2,3,6,7,8,8a-hexahydro-1,4-dioxo-	C_10_H_15_N_3_O_3_
20	71.968	1,388,758	13.44	Pyrrolo[1,2-a]pyrazine-1,4-dione, hexahydro-3-(phenylmethyl)-	C_14_H_16_N_2_O_2_

## Data Availability

The original contributions presented in this study are included in the article. Further inquiries can be directed to the corresponding authors.

## Abbreviations

The following abbreviations are used in this manuscript:

PTM	Potato tuber moth
EPN	Entomopathogenic nematode
IJs	Infective juveniles
ESB	Endosymbiotic bacteria
BAC	Bioactive compound
